# Exploring the research landscape of exosomes in Parkinson’s disease: a bibliometric analysis

**DOI:** 10.3389/fneur.2026.1880805

**Published:** 2026-08-25

**Authors:** Yuqi Liu, Yun Liu, Yujie Li, Yujie Wang, Jianhui Liu

**Affiliations:** 1 The Sixth Hospital of Guiyang, Guiyang, China; 2 The First Clinical Medical College of Guizhou University of Traditional Chinese Medicine, Guiyang, China; 3 School of Basic Medical Sciences, Zhejiang Chinese Medicine University, Hangzhou, China; 4 Weng'an County People's Hospital, Weng'an, Guizhou, China; 5 Department of Neurology, The Second Affiliated Hospital of Guizhou University of Traditional Chinese Medicine, Guiyang, China

**Keywords:** bibliometric analysis, biomarkers, exosomes, Parkinson’s disease, research trends

## Abstract

**Background:**

Parkinson’s disease (PD) is characterized by progressive loss of dopaminergic neurons in the substantia nigra and pathological aggregation of *α*-synuclein, while early diagnosis and disease stratification remain constrained by the lack of sensitive and specific biomarkers. Exosomes carry proteins, lipids, and diverse nucleic acids and mediate intercellular communication; they may participate in *α*-synuclein propagation and neuroinflammation and have become a research focus as biomarkers and delivery vehicles due to their stability and potential to cross the blood–brain barrier.

**Methods:**

Original articles and reviews published between January 1, 2005 and December 31, 2025 were retrieved from the Web of Science Core Collection (WoSCC) and Scopus; 1,486 publications were included after merging and deduplication. CiteSpace, VOSviewer, Bibliometrix, and Microsoft Excel were used for bibliometric and visualization analyses, covering publication output, contributing countries and institutions, authors, journals, citation counts, and keyword trends.

**Results:**

PD-related exosome research spanned 63 countries, with publication output accelerating markedly after 2019 and peaking in 2023 (231 articles), accumulating 72,631 citations. China (427 articles, 28.7%) and the United States (238 articles, 16%) were the leading contributors, with the United States exhibiting a more prominent hub role in international collaboration networks. Taipei Medical University and Capital Medical University were among the most productive institutions; Zhang, Jing, and Shi, Min were among the most influential authors. *International Journal of Molecular Sciences* served as a core publication venue. Keyword clustering revealed three main research lines: disease models and clinical context; biofluid-derived exosomal biomarkers and diagnostic evaluation; and mechanistic studies on *α*-synuclein propagation and neuroinflammation. Temporal evolution indicated a shift from mechanistic exploration toward clinical translation, with “engineered exosomes,” “delivery,” and “stem cell therapy” emerging rapidly in recent years.

**Conclusion:**

PD-related exosome research is undergoing sustained expansion with an increasingly translational orientation, and its focus shifting from pathological mechanisms toward biofluid biomarker development and engineered exosome-based delivery strategies. Mapping this knowledge landscape facilitates identification of key teams and emerging directions, providing a reference for methodological standardization, multicenter clinical validation, and interdisciplinary collaboration to advance clinical translation.

## 1 Introduction

Parkinson’s disease (PD) is the second most common neurodegenerative disorder worldwide, second only to Alzheimer’s disease, affecting more than 10 million individuals globally (1). It is characterized by progressive loss of dopaminergic neurons in the substantia nigra and pathological aggregation of *α*-synuclein (2). The incidence of PD increases markedly with advancing age, imposing a substantial and growing burden on global public health systems (3). The diagnosis of PD primarily relies on clinical manifestations, and current therapies are largely symptomatic, without the ability to halt or reverse neurodegeneration (4). Moreover, the lack of sensitive and specific biomarkers severely limits early detection, accurate disease stratification, and timely clinical intervention (5), posing major challenges to precision medicine strategies in clinical PD management.

**Figure 1:**
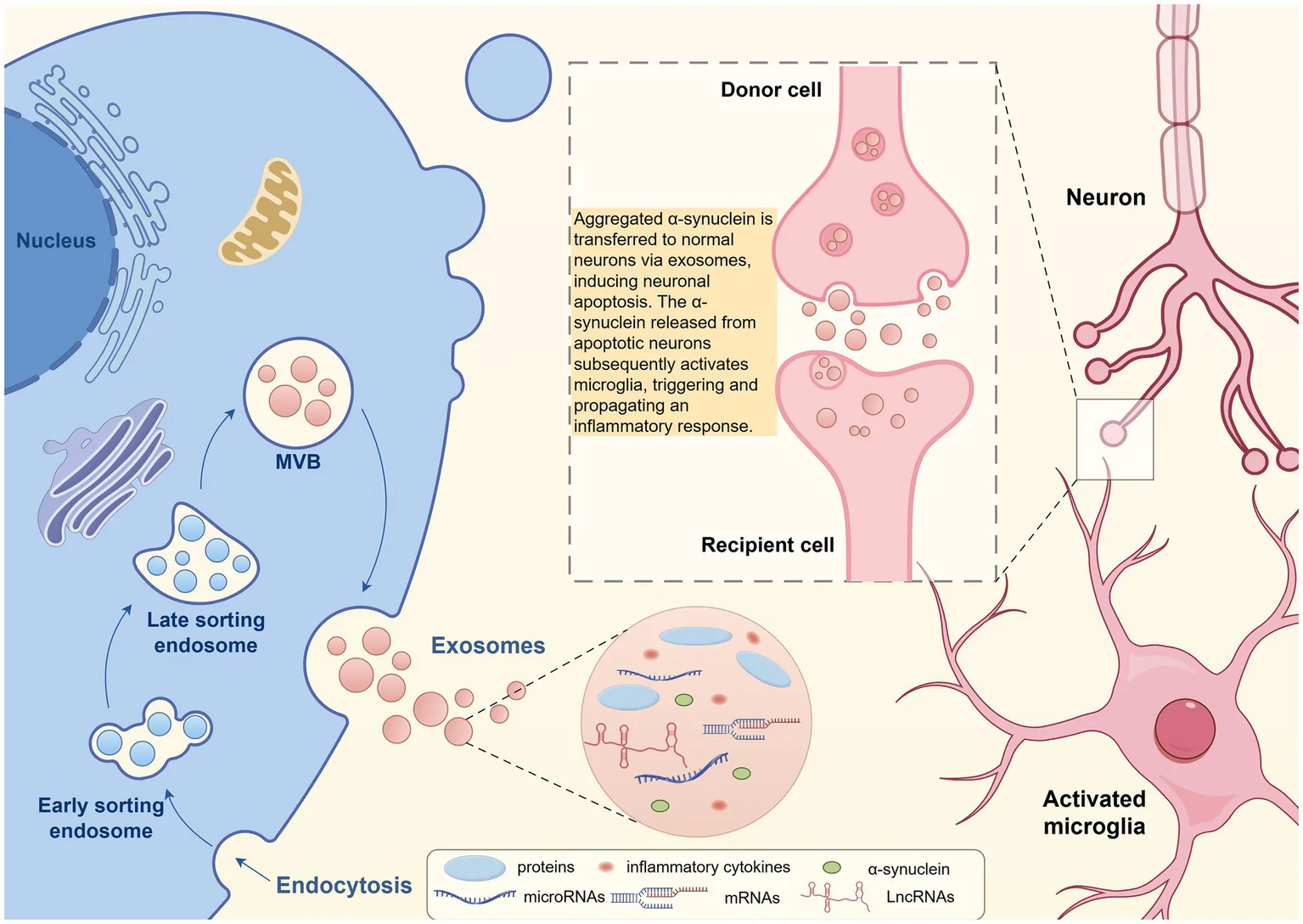
Disease regulatory mechanisms involving exosomes.

Exosomes are small membrane-bound vesicles 30–150 nm in diameter that are derived from multivesicular bodies and enriched in bioactive molecules, such as proteins, lipids, mRNAs, microRNAs, and long noncoding RNAs, thereby facilitating intercellular communication (6). Owing to their nanoscale size, stable lipid bilayer structure, and capacity to transport diverse bioactive molecules, exosomes have been extensively investigated in neurological disorders (7). Accumulating evidence indicates that exosomes participate in multiple pathological processes in PD, including the propagation of misfolded *α*-synuclein (8), promotion of neuronal apoptosis (9), and activation of neuroinflammation (10) (Figure 1). In addition, exosomes exhibit unique advantages in biomarker discovery and targeted therapeutic delivery (11). Existing PD studies have reported that exosomes mediate intercellular transfer of *α*-synuclein and may serve as potential carriers for targeted delivery of therapeutics to the central nervous system (12). Although several studies have explored the applications of exosomes in PD diagnosis, therapy, and targeted drug delivery, visualization analyses of application trends, leading authors, and research hotspots in PD-related exosome research remain insufficient (13).

Bibliometric analysis provides an objective and systematic approach to evaluating large-scale scientific literature, enabling the identification of influential contributors, collaboration networks, and emerging research themes (14). The rapidly growing body of literature on exosomes and PD reflects increasing interdisciplinary integration among neuroscience, molecular biology, and translational medicine (15). Meanwhile, comprehensive and quantitative evaluation of the knowledge structure, research hotspots, and developmental trends in this field has become particularly important (16). This study aimed to perform a systematic bibliometric analysis of English-language publications related to exosomes and PD from 2005 to 2025. By visualizing publication trends, patterns of international collaboration, and thematic evolution, this study provides data-driven insights into the knowledge structure and future research directions of this field.

## 2 Methods

### 2.1 Data sources

Data were obtained from the Web of Science Core Collection (WoSCC) and Scopus databases. WoSCC is a multidisciplinary citation database that provides comprehensive bibliographic records and structured citation information and has been widely used in bibliometric research (17). Scopus, maintained by Elsevier, offers broad journal coverage and detailed subject classifications, thereby complementing WoSCC in multidisciplinary literature analyses (18). Keyword selection was based on a broad definition of the field and relevant terminology, encompassing medical terms, Medical Subject Headings (MeSH) vocabulary, and disease-related synonyms. The search period spanned January 1, 2005 to December 31, 2025, and the bibliographic data were downloaded on January 6, 2026. The advanced search strategy was as follows: (“Exosome” OR “Exosomes” OR “Extracellular vesicle” OR “Extracellular vesicles” OR “EVs”) AND (“Parkinson” OR “Parkinson disease” OR “Parkinson’s disease”). Searches were conducted using the TS field in WoSCC and the TITLE-ABS-KEY field in Scopus. The detailed search strategy is presented in Supplementary Table 1. To ensure scientific quality and consistency of the included literature, only English-language original research articles and review articles were retained for analysis. Using this search strategy, 922 records from WoSCC and 1,381 records from Scopus were retrieved and downloaded.

### 2.2 Data selection and standardization

**Figure 2:**
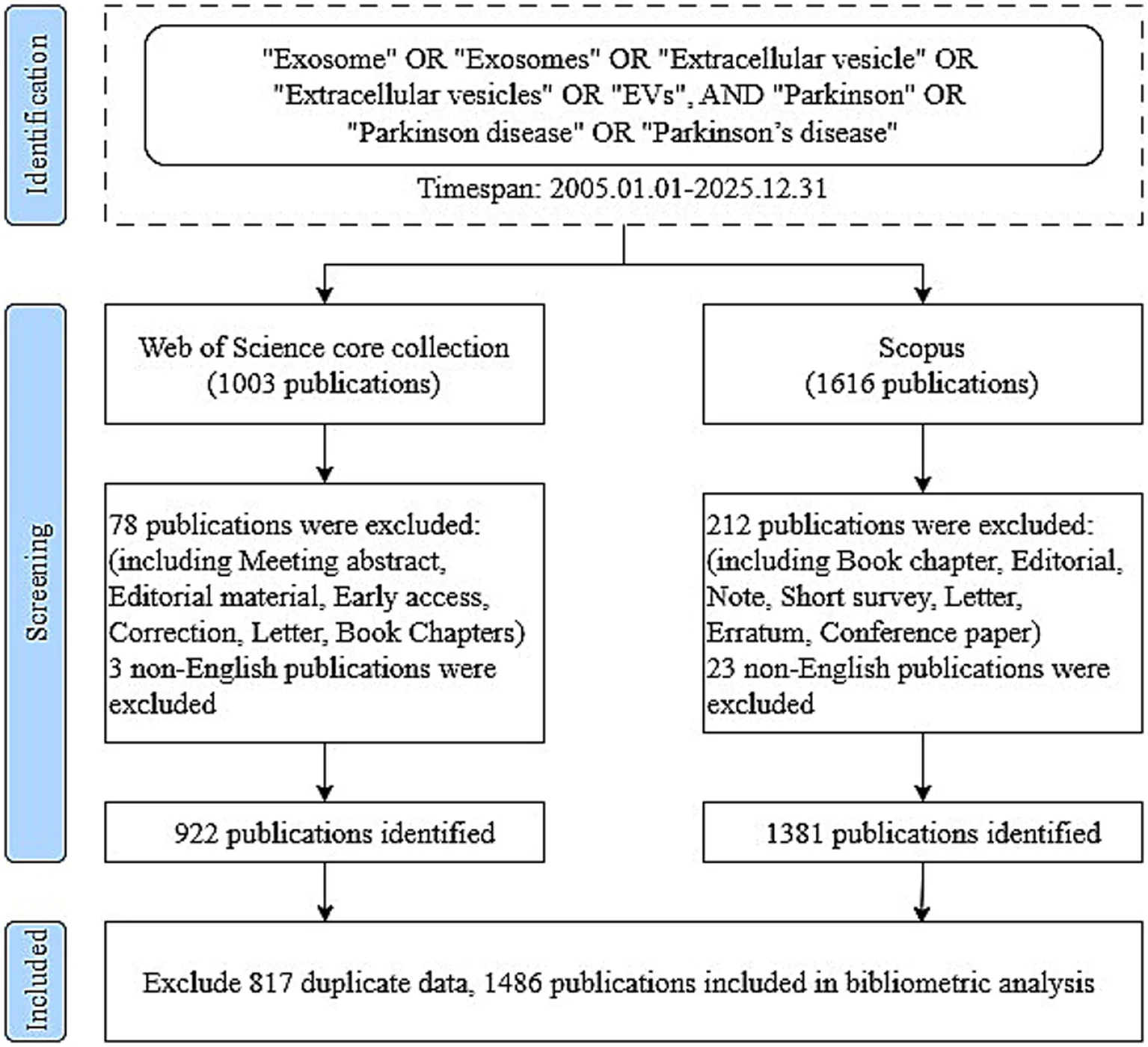
Flowchart of the literature screening process.

Bibliographic records retrieved from the WoSCC and Scopus databases were imported into NoteExpress for subsequent management, screening, and classification. Literature screening was initially performed based on titles and abstracts, with full-text assessment conducted when necessary. Studies were excluded if they did not simultaneously involve exosomes and PD, were unrelated to PD, or did not meet the predefined document type criteria. After records from the two databases were merged and duplicate publications were removed according to title, author, and year information, 1,486 eligible publications were ultimately included in the study and used for bibliometric analysis of the association between exosomes and PD. The literature screening process (Figure 2) was conducted independently by two researchers. In cases of disagreement during literature screening, a third researcher participated and verified the results. In addition, variations in abbreviations of the same author’s name or changes in the order of surname and given name were confirmed using ORCID information and author affiliations, and variants of institutional names were standardized to their full English names.

### 2.3 Bibliometric and visualization analyses

In this study, VOSviewer 1.6.20, CiteSpace 6.4. R1, and the R package bibliometrix (v4.5.0) were used to perform bibliometric analyses of literature related to exosomes and PD. Data visualization and figure formatting were primarily conducted using Microsoft Office Excel 2019 and SCImago Graphica (Beta 1.0.51). VOSviewer was used to process large-scale bibliometric data and to construct and analyze networks of countries/regions, institutions, authors, journals, highly cited publications, and keywords. CiteSpace was used to visualize knowledge maps and to identify research hotspots and potential emerging trends through citation analysis and burst detection; it was also applied to journal dual-map overlay analysis and reference citation burst detection. Bibliometrix was used to conduct systematic bibliometric statistical analyses and to support the construction and visualization of multiple types of networks and matrices. Core journals were defined based on descriptive statistical results for the distribution characteristics of publication output and citation frequency. Journal impact factor (IF) data were obtained from the 2025 edition of the Journal Citation Reports (JCR).

## 3 Results

### 3.1 Global distribution of publications and citation trends

**Figure 3:**
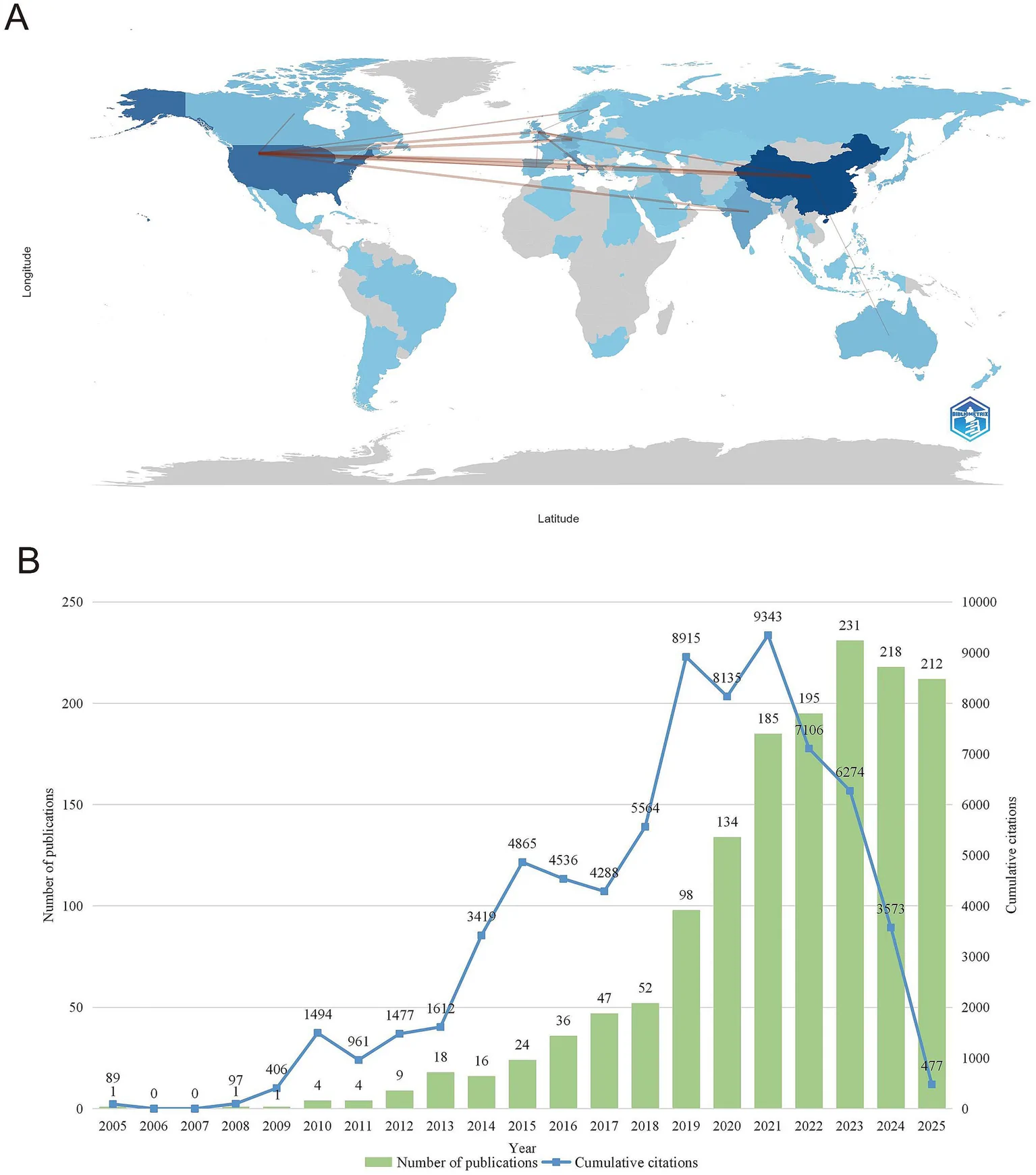
Global distribution of publications and citation trends. **(A)** Geographic distribution of PD-related exosome publications. **(B)** Publication trends in PD-related exosome research.

At present, research on exosomes in PD spans 63 countries worldwide, with most publications originating from East Asia, North America, and Western Europe (Figure 3A). In addition, Figure 3B depicts the annual publication trend for exosome-related PD research. During the early period (2005–2012), publication activity remained limited, with fewer than 10 articles published per year on average. From 2013 to 2018, research output increased steadily. Subsequently, from 2019 onward, the annual number of publications increased sharply and continued to grow, reaching a peak of 231 publications in 2023 and remaining at a high level in the following years. The 1,486 publications were cited 72,631 times in the databases. Between 2005 and 2021, the annual number of publications was positively correlated with total citations, with citations peaking in 2021. The year-by-year decline in total citations after 2022 may be attributable to delayed attention to newly published articles. Overall, these results indicate that this research field is in a phase of sustained growth.

### 3.2 Contributions of countries and institutions

**Figure 4:**
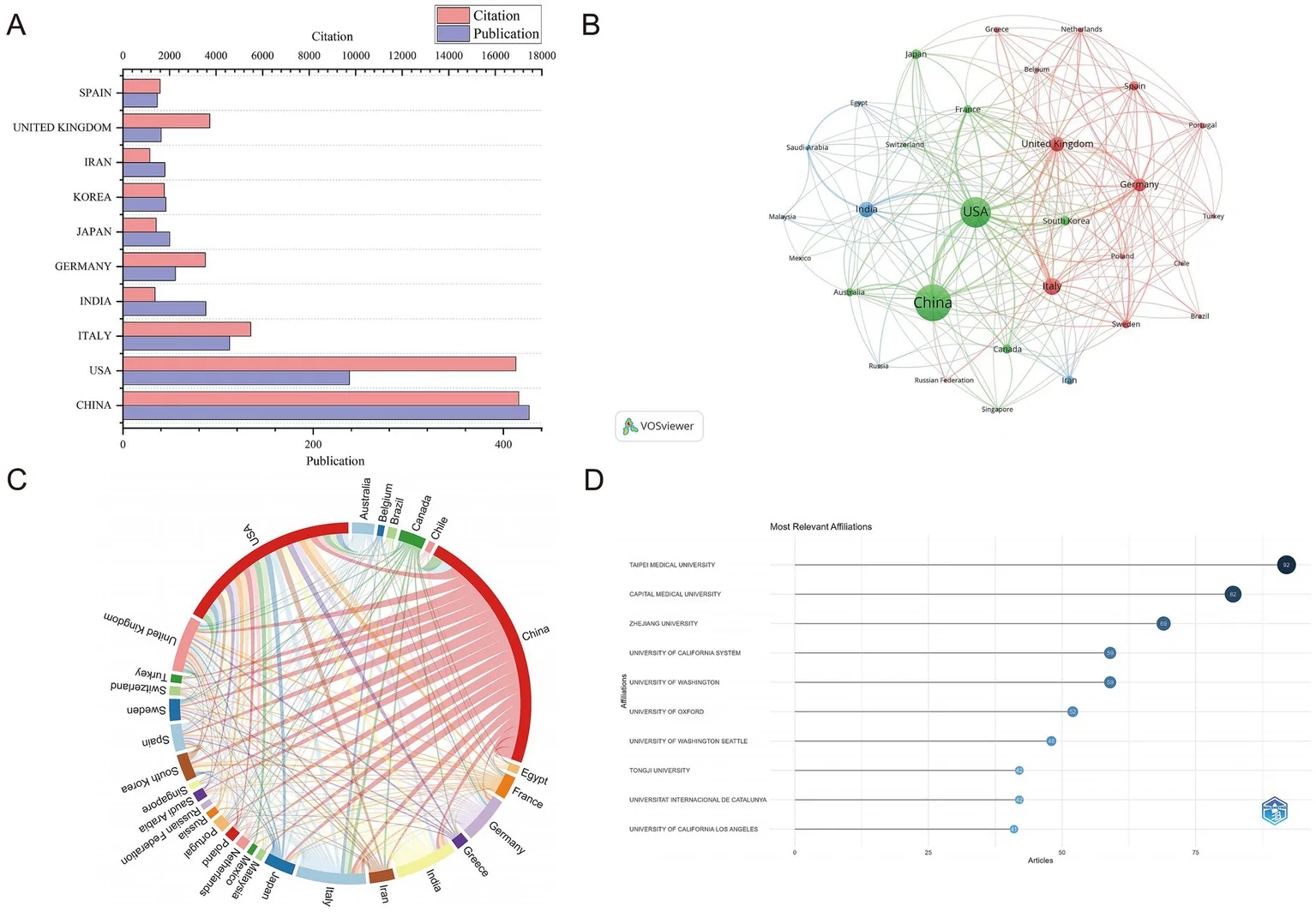
Distribution of publications across countries and institutions. **(A)** Publication output and citations of the top 10 countries. **(B)** Linkages among countries based on publication relationships. **(C)** Chord diagram of international research collaborations. **(D)** Publication output of the top 10 institutions.

Bibliometric analysis of publications on exosomes in the field of PD across different countries showed that the top 10 countries ranked by total publication output are listed in Table 1 and Figure 4A. China ranked first with 427 publications (28.7%), followed by the United States with 238 publications (16.0%). The remaining eight countries each contributed between 30 and 120 publications, indicating that China and the United States occupy leading positions in this field. In terms of total citations, China and the United States were comparable, with 17,004 and 16,873 citations, respectively, whereas most of the other eight countries accrued fewer than 5,500 citations. Regarding the proportion of international collaboration, the United Kingdom exhibited a relatively high collaboration rate (40%), suggesting its involvement in pivotal high-quality research outputs in this field.

**Table 1:** Top 10 countries by publications, SCP, MCP, and citations.

Rank	Country	Articles	SCP	MCP	MCP ratio (%)	Total citations	Average citations
1	China	427 (28.7%)	383	44	10.3	17,004	39.8
2	USA	238 (16%)	196	42	17.6	16,873	70.9
3	Italy	112 (7.5%)	91	21	18.8	5,483	49.0
4	India	87 (5.8%)	73	14	16.1	1,369	15.7
5	Germany	55 (3.7%)	42	13	23.6	3,536	64.3
6	Japan	49 (3.3%)	43	6	12.2	1,418	28.9
7	Korea	45 (3%)	43	2	4.4	1769	39.3
8	Iran	44 (3%)	38	6	13.6	1,148	26.1
9	United Kingdom	40 (2.7%)	24	16	40	3,724	93.1
10	Spain	36 (2.4%)	30	6	16.7	1,590	44.2

Subsequently, VOSviewer was used to perform inter-country collaboration analysis based on all publications from these 63 countries to characterize international cooperation. In the network map, larger circles indicate higher publication output, and links between countries reflect the strength of collaboration (Figure 4B). China and the United States represent the core forces in this research area. The United States not only produced a large volume of publications but also occupied the most central position in the network, acting as a bridge in international collaboration. The chord diagram of national collaboration shows that the linkage between China and the USA is the widest, forming the most prominent axis of international collaboration (Figure 4C). In addition, countries such as the United Kingdom, Italy, and Japan display numerous outward linkages, indicating active participation in multilateral research collaboration networks.

To investigate the contributions of 1,813 institutions identified in the field of exosome research in PD, we conducted a bibliometric analysis of the literature published by these institutions. As shown in Table 2 and Figure 4D, Taipei Medical University (92 articles) and Capital Medical University (82 articles) ranked first and second, with notably higher outputs than the third-ranked institution. In addition, prominent universities such as Zhejiang University (69 articles) and the University of Oxford (52 articles) also demonstrated high research activity. Overall, China and the United States occupy pivotal positions in this field.

**Table 2:** Top 10 institutions by publications.

Rank	Institution	Articles	Country/Region
1	Taipei Medical University	92	Chinese Taipei
2	Capital Medical University	82	China
3	Zhejiang University	69	China
4	University of California System	59	USA
5	University of Washington	59	USA
6	University of Oxford	52	UK
7	University of Washington Seattle	48	USA
8	Tongji University	42	China
9	Universitat internacional de Catalunya	42	Spain
10	University of California Los Angeles	41	USA

### 3.3 Contributions of authors and journals

**Figure 5:**
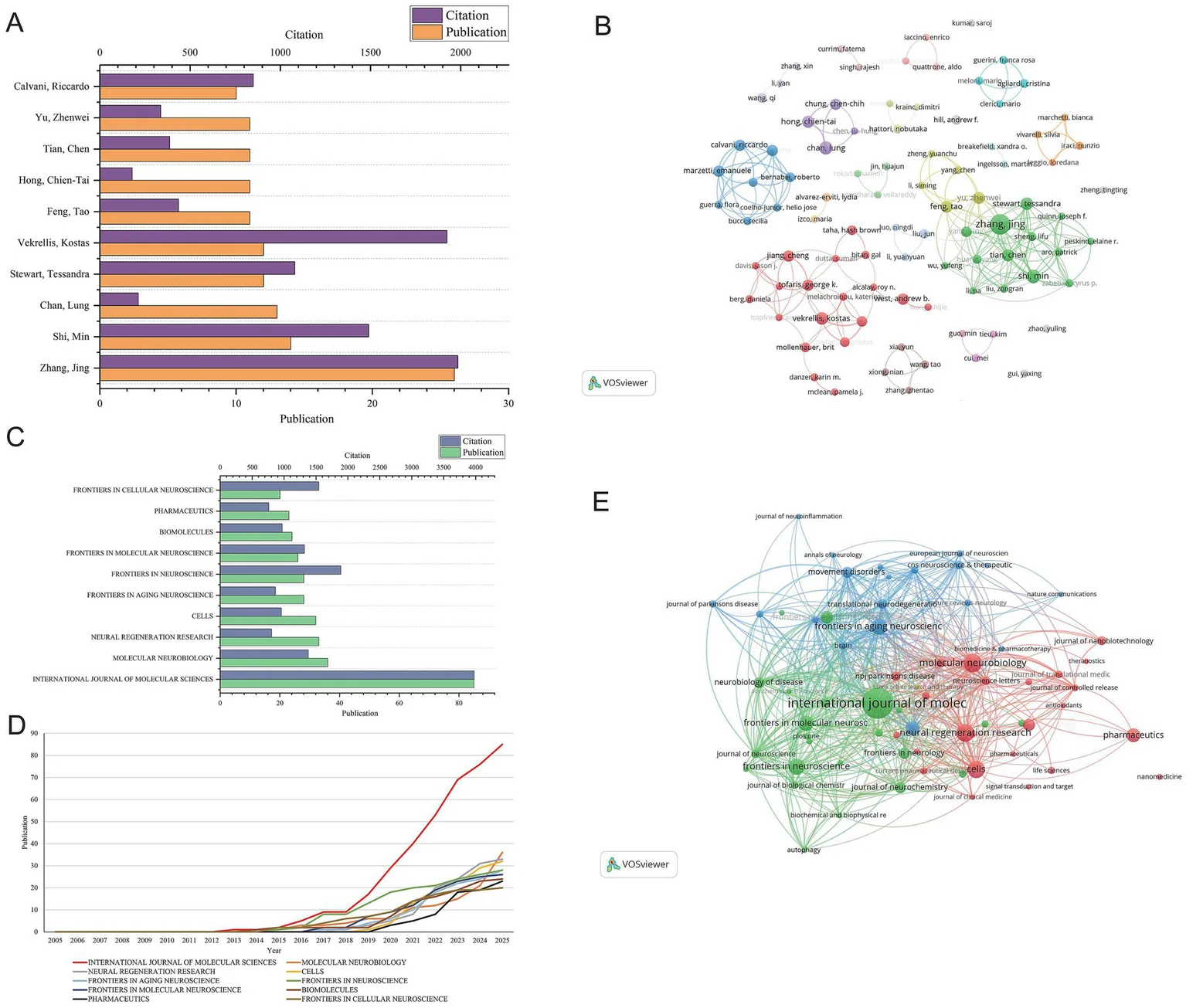
Visualization of authors and publishing journals. **(A)** Publication output and citations of the top 10 authors. **(B)** Author collaboration network in PD-related exosome research. **(C)** Publication output and citations of the top 10 journals. **(D)** Dynamic trends in annual publications of the top 10 journals. **(E)** Journal network map.

A total of 7,852 researchers have investigated exosomes and contributed to the field of PD. As shown in Table 3 and Figure 5A, Zhang, Jing (*N* = 26) and Shi, Min (*N* = 14) published the most papers, but their outputs did not differ substantially from those of the following eight researchers. In addition, Zhang, Jing (*N* = 1,983) and Vekrellis, Kostas (*N* = 1,923) each accrued more than 1,900 citations, indicating their foundational contributions to the field. VOSviewer was also used to visualize authors who published more than five papers in this domain (Figure 5B). Each cluster represents close collaborative relationships within the group. Based on the clustering results, Zhang, Jing, Feng, Tao, Jiang, Cheng, and Calvani, Riccardo may be among the most influential members within their respective international research communities.

**Table 3:** Top 10 authors by publication output and total citations.

Rank	Author	Publications	Total citations
1	Zhang, Jing	26	1983
2	Shi, Min	14	1,489
3	Chan, Lung	13	212
4	Stewart, Tessandra	12	1,080
5	Vekrellis, Kostas	12	1923
6	Feng, Tao	11	435
7	Hong, Chien-tai	11	178
8	Tian, Chen	11	386
9	Yu, Zhenwei	11	336
10	Calvani, Riccardo	10	850

A total of 1,486 publications related to exosomes and PD were distributed across 525 journals. Table 4 lists the top 10 journals ranked by publication output. *International Journal of Molecular Sciences* (*N* = 85) showed a pronounced lead in both publication output and total citations, suggesting that it serves as a core knowledge outlet in this niche field, whereas journals ranked second to tenth each published 20 to 36 articles (Figure 5C). These journals are well recognized in neuroscience, biomedicine, and multidisciplinary research. Figure 5D shows the temporal publication trends of major journals. After 2018, publication outputs across neuroscience and molecular biology journals increased markedly, with *International Journal of Molecular Sciences* exhibiting the steepest growth.

**Table 4:** Top 10 journalsby publications, H-index, IF, and JCR quartile.

Rank	Journal	Publications	Citations	H-index	IF	JCR
1	International Journal of Molecular Sciences	85	3,976	114	4.9	Q1
2	Molecular Neurobiology	36	1,380	96	4.3	Q1
3	Neural Regeneration Research	33	805	24	6.7	Q1
4	Cells	32	955	14	5.2	Q1
5	Frontiers in Aging Neuroscience	28	864	55	4.9	Q1
6	Frontiers in Neuroscience	28	1888	71	3.2	Q1
7	Frontiers in Molecular Neuroscience	26	1,317	46	3.8	Q1
8	Biomolecules	24	970	—	4.8	Q1
9	Pharmaceutics	23	759	32	5.5	Q1
10	Frontiers in Cellular Neuroscience	20	1,543	64	4	Q1

In the VOSviewer analysis, journals with at least five publications were included, yielding a journal co-occurrence network comprising 67 journals (Figure 5E). Each cluster represents a group of closely related journals in relevant fields. Among them, the green cluster was the largest and mainly included journals in fundamental molecular biology, including *International Journal of Molecular Sciences*, *Frontiers in Molecular Neuroscience*, and *Neurobiology of Disease.* The blue cluster primarily involved clinical neurology and aging research, with representative journals including *Frontiers in Aging Neuroscience* and *Movement Disorders*. The red cluster contained multiple nodes, including *Neural Regeneration Research*, *Molecular Neurobiology*, and *Cells*, reflecting comprehensive multidisciplinary journals.

### 3.4 Keyword co-occurrence and clustering analysis

**Figure 6:**
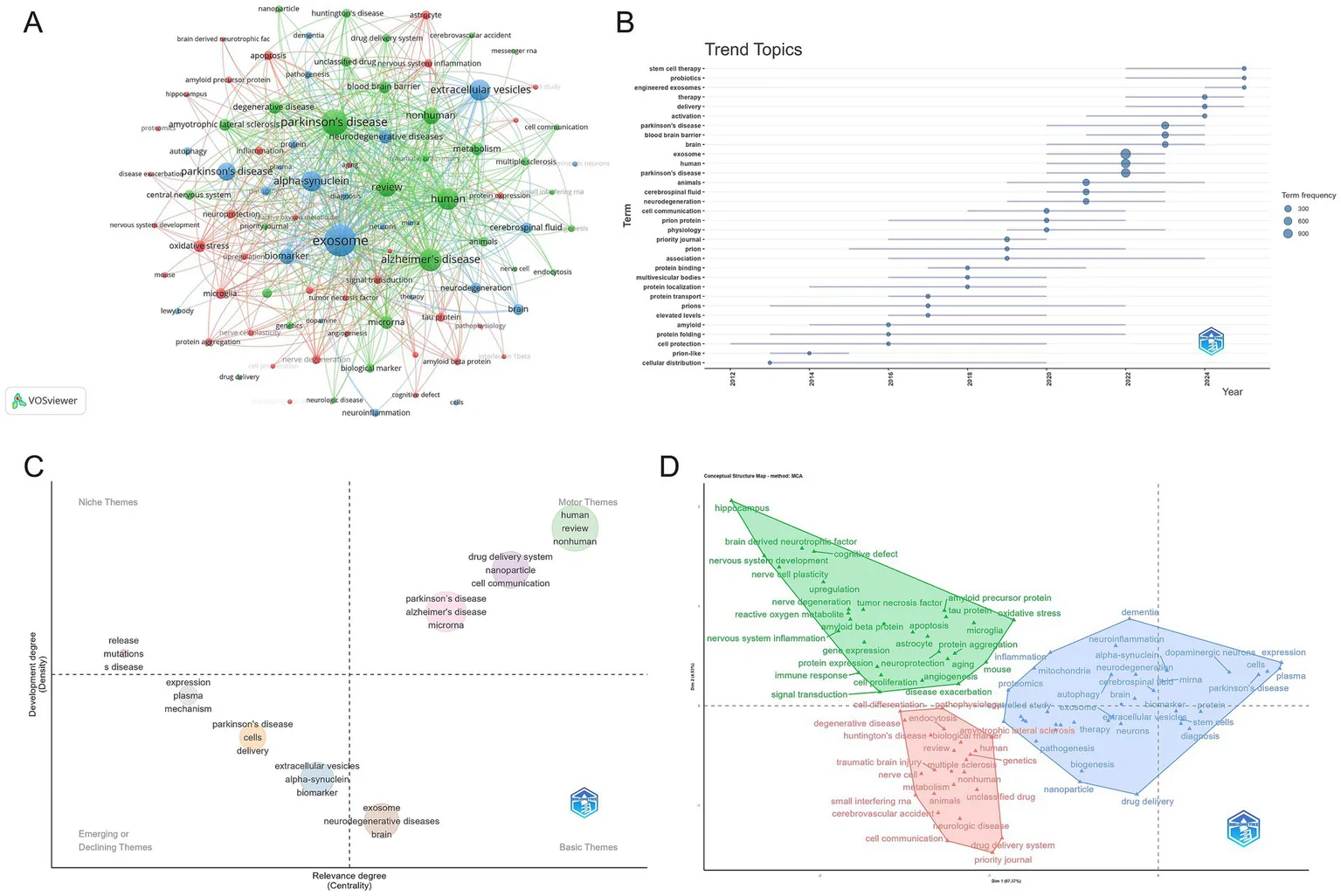
Keyword hotspots in PD-related exosome research. **(A)** Keyword co-occurrence network of PD-related exosome research generated by VOSviewer. **(B)** Trend analysis of keyword-related themes. **(C)** Thematic strategic map: the x-axis represents centrality and the y-axis represents density; Quadrant I indicates mature and central themes, Quadrant II indicates peripheral yet well-developed themes, Quadrant III indicates emerging or declining themes, and Quadrant IV indicates basic themes with cross-disciplinary supporting roles. **(D)** Visualization of the conceptual structure of keywords.

Keyword co-occurrence networks can visually reflect research hotspots and the knowledge structure of a specific field. In this study VOSviewer was used to construct a keyword co-occurrence map. Node size indicated that “exosome” and “extracellular vesicles” were the most intensively discussed hotspots in this field. Clustering analysis further partitioned the knowledge network of this field into three main research directions (Figure 6A). The green cluster represents macroscopic disease models and clinical context; the blue cluster emphasizes frontier diagnostics and target exploration focusing on the potential of exosomes and *α*-synuclein as novel biomarkers; and the red cluster concentrates on underlying pathophysiological mechanisms. In the temporal evolution of research themes (Figure 6B) the results suggest an evolutionary pattern basic mechanistic exploration toward targeted therapeutic translation. Since 2020 with the surge in research on biofluid biomarkers such as “exosome” and “cerebrospinal fluid,” field hotspots have gradually shifted toward clinical diagnosis and systematic evaluation. During 2023–2025 “engineered exosomes,” “delivery,” and “stem cell therapy” rapidly emerged as core hotspots indicating that precision therapeutic intervention using novel nanodelivery systems capable of traversing the blood–brain barrier (BBB) has become a current research frontier. A thematic map categorized research themes into four quadrants (Figure 6C) in which the motor themes quadrant aggregated “drug delivery system,” “nanoparticle,” “Parkinson’s disease,” and “microRNA,” potentially indicating that advanced nanocarrier-based delivery technologies are not only internally mature but also constitute a core engine driving the field forward. In contrast earlier themes such as “extracellular vesicles,” “biomarker,” and “expression” gradually declined in importance. The conceptual structure map revealed the knowledge framework of the field and visually delineated the intrinsic connections among its core concepts centered on the neuronal microenvironment, biomarkers, and systematic interventions in macroscopic disease models (Figure 6D).

### 3.5 Analysis of highly cited publications and references

**Figure 7:**
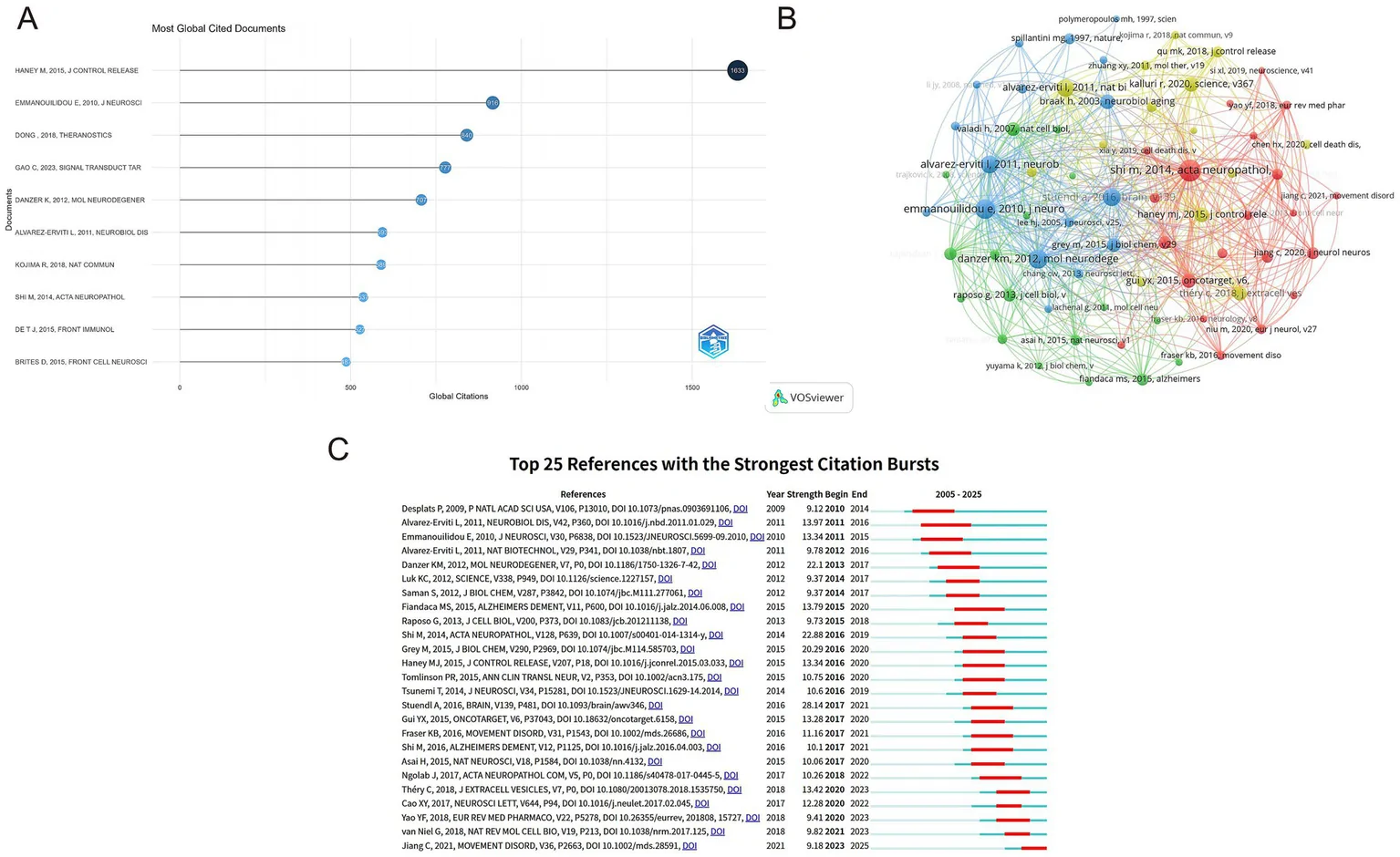
Highly cited references in PD-related exosome research. **(A)** Citation counts of the top 10 highly cited publications. **(B)** Co-citation network of PD-related exosome publications. **(C)** Top 25 most cited references identified by CiteSpace.

Figure 7A presents the top 10 most cited publications in this field, although highly cited publications are not necessarily high-quality publications. Among them, the article by Haney et al. (19), entitled “Exosomes as drug delivery vehicles for Parkinson’s disease therapy,” has been cited 1,633 times. This study emphasized that catalase-loaded exosomes delivered intranasally to PD mice exerted significant neuroprotective therapeutic effects in both *in vitro* and *in vivo* models. Other highly cited publications included Emmanouilidou et al. (20), Dong (21), and Gao et al. (22). Co-cited reference analysis of exosome-related literature in PD provides an important perspective for understanding the core publications in this field. Using VOSviewer to assess the interrelationships among these cited references, four major international collaboration clusters were identified (Figure 7B). Key references included studies by Shi et al. (2014) (51), Emmanouilidou et al. (20), and Alvarez-Erviti et al. (2011) (52). Burst analysis of co-cited references facilitates identification of emerging hotspots in PD exosome research and their duration (Figure 7C). The red segments indicate the time period during which a given co-cited reference remained in a citation burst, whereas the Strength value represents the citation burst intensity. Notably, most highly influential research outputs were published between 2014 and 2020. Among these, the study by Stuendl et al. (23), entitled “Induction of *α*-synuclein aggregate formation by CSF exosomes from patients with Parkinson’s disease and dementia with Lewy bodies,” was particularly notable; it reported that cerebrospinal fluid exosomes from patients with PD contain a pathogenic α-synuclein species that may initiate oligomerization of soluble α-synuclein in target cells, thereby triggering disease pathology.

## 4 Discussion

### 4.1 General information

The bibliometric findings of this study indicate that research on exosomes in PD has entered a phase of rapid expansion and follows a clear trajectory characterized by accelerated growth, hotspot differentiation, and translation-driven development. Globally, PD-related exosome research has involved 63 countries. However, publications are predominantly concentrated in East Asia, North America, and Western Europe, reflecting geographic clustering of resources and scientific output. At the national level, China ranked first with 427 publications (28.7%), followed by the United States with 238 publications (16%), together constituting the principal engines of research productivity in this field. Although China leads in publication volume, it remains behind the United States in citation impact and the overall strength of research institutions. Among the 1,813 institutions included, Taipei Medical University and Capital Medical University ranked first and second, and Chinese institutions such as Zhejiang University and Tongji University also performed prominently. Among US institutions, the University of California System and the University of Washington ranked among the leading contributors, and the University of Oxford in the United Kingdom also maintained high activity. This pattern closely aligns with the national distribution of research output. At the author level, although leading authors had higher publication outputs, no clear gap was observed, suggesting that PD-related exosome research is characterized by parallel contributions from multiple research teams, with knowledge contributions tending toward distributed accumulation. The VOSviewer author clustering network further indicated close collaboration within research communities and relatively clear thematic boundaries among communities.

The journal distribution showed that this research topic has a pronounced interdisciplinary spillover effect, covering multiple fields, including neuroscience, molecular biology, cell biology, and translational medicine. International Journal of Molecular Sciences ranked first with 85 publications and 3,976 citations. In keyword co-occurrence analysis, “exosomes” and “extracellular vesicles” encompassed the definition and classification of exosomes. EVs refer to vesicles with a bilayer membrane structure that are secreted by cells or shed from the plasma membrane (24), and are classified according to size into microvesicles, exosomes, and apoptotic bodies. However, as a subtype of EVs, exosomes are currently the most extensively studied, and in some articles, the term EVs refers only to exosomes. Clustering analysis showed that, since 2020, studies related to biofluid biomarkers such as “exosome” and “cerebrospinal fluid” have increased markedly, suggesting that the academic community has begun to explore more systematically the utility of exosomes in early diagnosis, disease staging, and therapeutic monitoring. During 2023–2025, “engineered exosomes,” “delivery,” and “stem cell therapy” rapidly emerged as core hotspots, indicating that engineered exosomes, as nanodelivery systems capable of crossing the BBB and enabling precise intervention, are becoming an emerging research frontier. Most of these papers focused on the biological properties and physiological functions of exosomes, as well as preclinical evaluation of their therapeutic potential in PD. For example, Haney et al. (19) provided experimental evidence supporting the ability of engineered exosomes to cross the BBB and deliver therapeutic molecules. Emmanouilidou et al. (20) found that α-synuclein can be secreted through a calcium-dependent exosomal pathway, which may represent an important route for the amplification and propagation of PD-related pathology between neurons. The review by Dong (21) covered brain drug delivery strategies over the previous 5 years and emphasized that BBB disruption and regulatory mechanisms are critical for designing effective delivery systems. In addition, representative high-impact publications published after 2019 appear to have further consolidated this developmental trajectory. The review by Kalluri and LeBleu (25), published in *Science*, comprehensively detailed the biological significance of exosomes in intercellular communication and disease progression, promoting a conceptual shift from viewing exosomes as passive carriers to recognizing their active regulatory roles. Welsh et al. published MISEV2023, the latest version of the widely accepted MISEV guidelines in the EV field, which is critical for discussing methodological standardization in exosome research and provides more detailed specifications for EV isolation, characterization, molecular marker quantification, and functional validation (26). Meanwhile, Gao et al. (22) integrated research directions related to microglial activation, neuroinflammatory signaling pathways, and therapeutic targets in PD, thereby becoming an important node in the evolving knowledge structure of this stage. Taken together, these bibliometric features suggest that PD-related exosome research has gradually shifted from basic descriptive studies toward mechanistic investigation.

### 4.2 Hotspots and frontiers

Co-occurrence analysis and burst analysis of keywords and co-cited references are important tools for tracking the evolution of disease-related research hotspots and emerging frontiers (27). Although keyword analysis indicates increasing attention to these areas, bibliometric methods cannot determine their future scientific significance or clinical success. In this study, PD-related exosome research can be broadly categorized into three main trajectories. The first trajectory involves the discovery and validation of high-quality biomarkers, with an emphasis on exploring their clinical value in early diagnosis, differential diagnosis, disease-course stratification, and therapeutic monitoring. The second trajectory focuses on mechanistic elucidation of the role of exosomes in PD pathophysiology. The third trajectory concerns the development of therapeutic exosomes and engineered delivery systems. Notably, these three trajectories are not independent of one another: mechanistic studies a provide biological rationale for biomarkers and therapeutic targets; biomarker studies, in turn, can be used to evaluate therapeutic delivery and interventional efficacy; and advances in engineered delivery may generate new mechanistic questions and new biomarker requirements.

#### 4.2.1 Impact of exosomal biomarkers in PD

Exosomes can carry misfolded *α*-synuclein and transfer it between cells, thereby promoting PD progression. They can be isolated from biological fluids such as blood and cerebrospinal fluid, making them valuable biomarkers for this disease (28). Exosomes have been shown to cross the blood–brain barrier and deliver miRNAs, proteins, and lipids to target cells (29). In PD models, exosomes derived from activated microglia or injured neurons are enriched in miR-155 and can activate inflammatory responses in recipient glial cells and neurons (30). In addition, neuronal exosomal miR-30a-5p and miR-181c-5p are downregulated under PD-related stress conditions, whereas supplementation of these miRNAs can alleviate mitochondrial dysfunction and cell death, suggesting that exosomal cargo may participate in the regulation of PD pathology (31). A clinical study found that serum L1 cell adhesion molecule (L1CAM)-positive extracellular vesicle-associated *α*-syn could distinguish individuals at high risk of or with prodromal PD from healthy controls, with a higher recognition rate among phenoconverters and a reported area under the curve of approximately 0.90 (32). In addition to α-syn, other miRNAs, such as miR-24, miR-151a-5p, and miR-214, have also been identified in serum- and cerebrospinal fluid-derived exosomes from patients with Parkinson’s disease (33). This has led to the gradual deepening of research on exosomal biomarkers for the diagnosis of PD.

#### 4.2.2 Interventional effects of exosomes in PD disease models

PD disease models are induced by neurotoxins such as 6-hydroxydopamine or 1-methyl-4-phenyl-1,2,3,6-tetrahydropyridine (MPTP), or generated through genetic modification, and can reliably reproduce key features of human Parkinson’s disease, including dopaminergic neuronal loss, motor dysfunction, and *α*-synuclein aggregation (34). In recent years, the role of exosomes in PD models has received increasing attention because of their involvement in intercellular signaling and pathological protein transport. Exosomes exhibit dual functions in the pathogenesis of PD: they can propagate disease through toxic substances while also exerting neuroprotective effects through specific molecules such as adenine (35). Experiments have shown that injection of exosomes derived from the serum of patients with PD or from oxidative stress-treated cells into the rat brain can induce α-synuclein aggregation, neuroinflammation, and neuronal apoptosis (36). Conversely, therapeutic applications using exosomes have shown promising potential in PD models. Rabies virus glycoprotein (RVG)-modified exosomes loaded with miRNAs, antioxidants, or neurotrophic factors and delivered via intranasal or intracerebroventricular routes can effectively target the brain, improve motor function, and reduce inflammation (37). In addition, exosomes released by activated microglia exacerbate dopaminergic neurotoxicity; inhibiting exosome release or altering their contents may provide new therapeutic strategies (38). As research progresses from modeling to mechanistic exploration and translational application, disease models are playing an increasingly important role in validating exosome-based interventions for PD.

#### 4.2.3 Exosome-based targeted therapeutic delivery systems

At present, there is no curative therapy for Parkinson’s disease. Existing treatments mainly alleviate symptoms but cannot effectively halt neurodegenerative pathology (39). Advances in nanomedicine and targeted delivery systems have made exosomes highly promising therapeutic platforms because of their endogenous origin, biocompatibility, and ability to penetrate the blood–brain barrier (40). Relevant studies have found that mesenchymal stem cell (MSC)-derived exosomal nanomedicines co-loaded with quercetin and the phosphorus dendrimer AK76 can treat PD by modulating the inflammatory–immune microenvironment (41). Engineered exosomes can be loaded with drugs, miRNAs, or siRNAs and delivered via intranasal, intravenous, or intracerebroventricular routes, exerting neuroprotective effects in PD models (42). Related studies have designed engineered extracellular vesicle nanodrug formulations to enhance therapeutic efficacy in PD through the dual perspectives of alleviating inflammation and regulating immune homeostasis (43). Studies have shown that exosomes derived from MSCs or neural stem cells (NSCs) can inhibit *α*-synuclein aggregation, reduce microglial activation, and improve motor function (44). Human umbilical cord MSC-derived exosomes exert neuroprotective effects by inducing autophagy, reducing dopaminergic neuronal loss and apoptosis, and increasing striatal dopamine levels (9). In addition, exosomes can regulate mitochondrial function, alleviate oxidative stress, and activate the autophagy–lysosomal pathway, thereby potentially triggering endogenous neuronal repair processes and slowing disease progression at the mechanistic level (45–47). Emerging strategies include modifying the exosome surface with the brain-targeting peptide RVG to improve delivery efficiency and therapeutic precision (48, 49). Synthetic nanovesicles that mimic the structure of exosomes have attracted considerable attention because of their higher yield and stability, while also showing similar neuroprotective potential in drug delivery systems (50). These advances indicate that exosomes are currently in the stage of preclinical validation and may provide multifaceted strategies for precision therapy in Parkinson’s disease in the future.

## 5 Limitations

The present findings also underscore intrinsic limitations of bibliometric analysis. First, data sources and search strategies may introduce coverage bias, particularly leading to underestimation of non-English literature, regional journals, or outputs not fully indexed by the databases. Second, citation counts are influenced by publication time, disciplinary citation practices, hotspot effects, and self-citation, and therefore cannot be equated with true scientific quality. Third, this study mainly relied on publication output and citation counts, and bibliometric indicators reflect influence and visibility rather than scientific validity. Fourth, the decline in total citations after 2022 is likely attributable to citation lag and truncation effects and should not be simplistically interpreted as a decrease in academic attention. Despite these limitations, this study delineates, at a macroscopic level, the growth drivers, global collaboration patterns, and thematic evolution pathways of PD-related exosome research, providing an actionable reference framework for topic planning, collaboration strategies, and translational route design.

## 6 Conclusion

In summary, based on English-language publications indexed in WoSCC and Scopus from 2005 to 2025, this study conducted systematic bibliometric and visualization analyses using VOSviewer, CiteSpace, and bibliometrix, and constructed a knowledge map of PD-related exosome research. The results indicate that this field has entered a phase of rapid expansion since 2019, with China and the United States serving as core contributors and key hubs of international collaboration. Research themes have shifted from early mechanistic exploration focused on *α*-synuclein propagation and neuroinflammation toward the development of biomarkers derived from cerebrospinal fluid or peripheral biofluid exosomes, and have further converged in recent years on translational frontiers such as engineered exosome-based BBB delivery, optimization of drug delivery systems, and stem cell-related therapies.

Future studies are expected to prioritize broader disease coverage, accumulation of high-quality clinical evidence, and establishment of standardized frameworks for exosome preparation and functional evaluation, while strengthening multidisciplinary collaboration to facilitate the clinical application of engineered exosomes. Overall, the bibliometric analysis presented herein provides foundational insights into the developmental trajectory and emerging frontiers of exosome research in neurodegenerative diseases, and establishes a strategic basis for future breakthroughs in diagnosis, targeted therapy, and clinical translation.

## Data Availability

The original contributions presented in the study are included in the article/Supplementary material, further inquiries can be directed to the corresponding author.
