# Supplementary material for: Exploring the research landscape of exosomes in Parkinson’s disease: a bibliometric analysis
Source: Front Neurol. 2026 Aug 25;17:1880805. doi: 10.3389/fneur.2026.1880805 (PMC13549832; doi:10.3389/fneur.2026.1880805)
Supplement: Supplementary file 2 [file Table_1.DOC]

Supplementary Material

**Supplemental Table 1.**Search strategy in the WoSCC and Scopus databases.

WoSCC: TS = ("Exosome" OR "Exosomes" OR "Extracellular vesicle" OR "Extracellular vesicles" OR "EVs") AND TS = ("Parkinson" OR "Parkinson disease" OR "Parkinson’s disease")

Scopus: TITLE-ABS-KEY ("Exosome" OR "Exosomes" OR "Extracellular vesicle" OR "Extracellular vesicles" OR "EVs") AND TITLE-ABS-KEY ("Parkinson" OR "Parkinson disease" OR "Parkinson’s disease")

**Note:** Add double quotes "" to qualify the search term as an exact search
